# Soft Foil

**Published:** 1881-02

**Authors:** 


### SOFT FOIL.

"At the last meeting of the American Dental Association, Dr. Joshua Tucker, of Boston, consented to deliver an address upon 'The Working Properties of Soft Foil,' and his very sensible remarks indicate a tendency of the leading minds in our profession to 'first principles.' He said that he believed the old system of filling teeth with soft gold to be the true and scientific way. He had watched with great interest the changes that had been made in the profession, and had heard many discussions on the subject of cohesive gold, and on its first introduction with the mallet he condemned it. He maintained all along that the principle was wrong. It was true that the cohesive gold would make a more beautiful filling and a better surface, but these things were not all that were wanted. Teeth decayed internally; there was a circulation going on through the dentine continually, and the mistake was made by pounding gold against the walls of the teeth that circulation was stopped. It was often found in three or four years that the tooth turned blue. That was owing to the circulation at the back of the gold, which caused the blueness and produced decay. By the old method it was never sought to make the gold solid against the wall or on the floor of the cavity, but to press the particles of soft gold against the wall till the cavity was completely filled. In his judgment the mistake made in filling teeth in the present day was from not stopping the tubuli — not damning them up. If there was anything true and scientific in the principle of the old method, the nearer they drew to it the better it would be for the profession."

The above, taken from *The Dental News*, published by Dr. T. P. Wagoner, of Knightstown, Ind., with not only approval but with commendation, would strike one as rather singular.

What is to be understood by the expression "his very sensible remarks indicate a tendency of the leading minds in our profession to 'first principles'"? What do you mean by first principles? Is it first in time or first in importance and value? What were the great and important and recognized principles of dental science forty years ago, to which all ought to return? Is there a dentist anywhere, who knows what the dental science and art of to-day is, who desires to return to these "first principles," when "it was never sought to make the gold solid against the wall or floor of the cavity," and still, notwithstanding this, "the particles of soft gold were pressed against the wall till the cavity was completely full." Now, to an ordinary comprehension, there would seem to be a contradiction in these quotations; but then, of course, there is not, for Bro. W. says they are very sensible remarks.

And then, what about "the circulation at the back of the gold, which caused the blueness and produced decay"? That was a very bad kind of circulation, indeed — yes, wicked. "The mistake in filling teeth in the present day is from not stopping the tubuli — not damning them up." Was the tubuli to be stopped by not making the gold solid against the wall or on the floor of the cavity? About one half of the statements in the above extract contradict the other half.

Dr. Tucker could not have put together such a batch of nonsense had he tried. He ought to prosecute the reporter for libel or slander, or ——.

Are we to turn our backs upon all the discoveries, improvements and progress made in the last forty years, and go to "first principles?" There have always been some who wanted to go back. When Israel was on the way to the promised land, some, yes, a great many of the "leading minds" wanted to go back to Egypt, to the fleshpots and to bondage.

